# Cerebral palsy characteristics in term‐born children with and without detectable perinatal risk factors: A cross‐sectional study

**DOI:** 10.1111/dmcn.16111

**Published:** 2024-10-15

**Authors:** Kai Suzuki, Nafisa Husein, Maryam Oskoui, Darcy Fehlings, Michael Shevell, Adam Kirton, Mary J. Dunbar, The Canadian Cerebral Palsy Registry, John Andersen, David Buckley, Darcy Fehlings, Lee Burkholder, Adam Kirton, Louise Koclas, Nicole Pigeon, Ram Mashal, Jordan Sheriko, Ellen Wood, Michael Shevell

**Affiliations:** ^1^ Department of Neuroscience University of Calgary Calgary AB Canada; ^2^ Research Institute‐McGill University Health Centre Montreal QC Canada; ^3^ Departments of Pediatrics and Neurology/Neurosurgery McGill University Montreal QC Canada; ^4^ Bloorview Research Institute, Department of Paediatrics University of Toronto ON Canada; ^5^ Alberta Children's Hospital Research Institute University of Calgary Calgary AB Canada; ^6^ Departments of Pediatrics University of Calgary Calgary AB Canada; ^7^ Hotchkiss Brain Institute Canada. 3330 Hospital Dr NW Calgary AB Canada; ^8^ Department of Community Health Sciences University of Calgary AB Canada

## Abstract

**Aim:**

To compare, in term‐born children with cerebral palsy (CP), the characteristics of those who exhibit detectable risk factors for CP at birth with those who do not.

**Method:**

This was a cross‐sectional study of term‐born children using the Canadian Cerebral Palsy Registry comparing those with and without perinatal risk factors and/or neonatal symptoms for pregnancy, birth and neonatal characteristics, magnetic resonance imaging (MRI) findings, CP subtype, and impairment severity. Risk factors were quantified with a CP risk calculator. Multivariable and multinomial regressions were expressed as odds ratios (OR) and relative risk ratios.

**Results:**

Of 1333 term‐born children, 781 (58.6%) had complete variables for the CP risk calculator, of whom 195 (25%) had ‘undetectable’ newborn infant CP risk, and they did not have greater postneonatal brain injury. Focal injury on MRI was more common (OR 2.0, 95% confidence interval [CI] 1.3–3.1) than in the ‘detectable’ group. The ‘undetectable’ group had more unilateral CP (OR 1.8, 95% CI 1.3–2.6), less severe motor impairment (OR 0.76, 95% CI 0.67–0.86), and were more verbal (OR 2.3, 95% CI 1.5–3.6).

**Interpretation:**

In the Canadian CP Registry, one‐quarter of term‐born children lacked neonatal encephalopathy, seizures, or perinatal risk factors. They were more likely to have unilateral CP, focal MRI findings, and communicate with words than children with risk factors or neonatal symptoms.

## Graphical abstract

This paper uses a previously published objective newborn infant cerebral palsy (CP) risk calculator and neonatal symptoms (encephalopathy or seizures) to identify participants in the Canadian CP Registry who have ‘undetectable’ CP risk at birth, and determine their phenotype to aid in earlier detection. Those with ‘undetectable’ risk are more likely to be female, have focal injury on MRI, unilateral CP, be ambulatory, and communicate with words. Early handedness before the age of 1 year should prompt referral for evaluation of CP, even in the absence of perinatal risk factors.

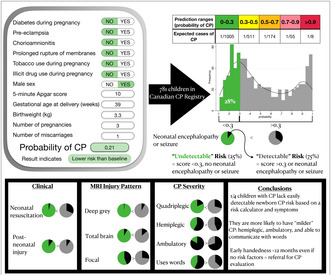

This original article is commented by Hoei‐Hansen et al. on pages 423–424 of this issue.

Plain language summary: https://onlinelibrary.wiley.com/doi/10.1111/dmcn.16154

**Abbreviation**
CCPR: Canadian Cerebral Palsy Registry.

**What this paper adds**

- ‘Detectable’ perinatal risk factors were absent in 25% of term‐born children in the Canadian Cerebral Palsy (CP) Registry.
- Focal brain injury and hemiparesis were more common in children without detectable CP risk factors.
- Those without detectable risk factors were more likely to be ambulatory and communicate with words.
- Screening for early handedness may be most practical for early detection.

Cerebral palsy (CP) is a lifelong motor impairment caused by non‐progressive disturbance of early brain development, and is the most common physical disability of childhood.^1^ CP provides a label for the clinical manifestations of various brain conditions and can be categorized by phenotype as spastic (unilateral and bilateral), dyskinetic, hypotonic, and ataxic.^2^ The range of motor function can be described using scales such as the Gross Motor Function Classification System (GMFCS) or Manual Ability Classification System. In addition to motor challenges, children with CP can have impairments in cognition, vision, hearing, and communication.^3^

In high‐resource settings the estimated incidence of CP is 1.6 per 1000 births,^4^ which may be declining.^5^, ^6^ Approximately 90% to 94% of CP is attributed to the antenatal and neonatal periods; and 6% to 10% to the postneonatal period.^7^, ^8^, ^9^, ^10^ Historically, CP was particularly associated with preterm birth and hypoxic–ischemic encephalopathy, both of which are evident in neonates and often require care in a neonatal intensive care unit.^11^ Early diagnosis of CP is facilitated by follow‐up programs in neonatal intensive care units using tools such as the General Movements Assessment or specific magnetic resonance imaging (MRI) biomarkers.^12^ Early screening can reduce the time to diagnosis from 12 to 18 months of age to 6 months of age.^11^ After CP is diagnosed, infants can be engaged in targeted, evidence‐based therapies to improve their outcomes.^13^

However, approximately 40% of children with CP do not undergo early screening because they do not have neonatal symptoms or are not admitted to a neonatal intensive care unit, and their CP diagnosis is delayed by months or years.^14^, ^15^ To address this gap, a recent study used the Canadian Cerebral Palsy Registry (CCPR) and a large group of typically developing comparison children to determine whether combinations of risk factors could help predict CP risk in newborn infants at term.^16^ The authors of the study developed a risk calculator using 12 clinical variables usually available at birth, the output of which is a score from 0 to 1. Most of the comparison children (70%) had scores below 0.3, which was proposed as a cut‐off for CP screening with a sensitivity of 65% (95% confidence interval [CI] 60–68) and specificity of 71% (95% CI 69–73). However, 28% of the children with CP also had scores below 0.3, and thus the risk calculator would not identify them for early screening, early diagnosis, or therapy. To achieve earlier CP diagnosis in this subset of term‐born children, an understanding of their phenotype for CP type, clinical findings, severity, imaging findings, and other variables is needed.

The aim of the present study was to compare characteristics of CP in children born at term with and without detectable perinatal risk factors as determined by a validated CP risk calculator^16^ and/or neonatal symptoms such as encephalopathy or seizures, who were born at term. We hypothesized this group may have had more postneonatally acquired CP.

## METHOD

### Study design

This was a cross‐sectional study using the CCPR.

### Setting

The CCPR was founded in 2003 in Quebec and expanded to Northern Alberta (2007), British Columbia (2011), Manitoba (2017), Newfoundland (2011), Nova Scotia (2011), and the Greater Toronto Area of Ontario (2010). Children with CP were recruited from pediatric rehabilitation centers and hospitals from 2003 onwards. Data were collected through in‐person or telephone interviews with parents/guardians as well as medical chart review. All children in the CCPR were recruited at 2 years of age or older.^17^ CP was diagnosed as previously described,^18^ and confirmed at the age of 5 years. Ethics approval for the CCPR was obtained from the Research Institute of the McGill University Health Center (Research Ethics Board number 2010–398) and from each center. Written informed consent was obtained from a caregiver. The data were received as a de‐identified data set. All participants in the study were evaluated using the same definitions and measurements (Table S1) performed by local pediatric neurologists and developmental pediatricians. Results are reported according to the Strengthening the Reporting of Observational Studies in Epidemiology (STROBE) checklist.^19^

### Participants

We included participants born from 2003 to 2018 with a gestational age of 37 weeks or greater at birth with complete variables for the CP risk calculator. The 12 variables required to be complete were number of previous pregnancies and miscarriages, diabetes, preeclampsia, chorioamnionitis, maternal tobacco or illicit drug use, prolonged rupture of membranes, 5‐minute Apgar score, gestational age at delivery, birthweight, and infant sex. The definitions for these variables are provided in Table S1. All participants were also included in a previous study for the development of a CP risk calculator.^16^

### Variables

Variables are defined in Table S1. The following pregnancy‐related variables were collected: physical trauma during pregnancy, gestational diabetes, preeclampsia, chorioamnionitis, twin pregnancy, exposure to tobacco/drugs/alcohol, vaginal bleeding, maternal fever during delivery, and intrauterine growth restriction (all binary yes/no), and the number of pregnancies and miscarriages (continuous). Delivery variables were presence of maternal fever, need for resuscitation, emergency Caesarean section, and prolonged rupture of membranes (all binary). Infant variables included sex (binary male/female), and birthweight, gestational age at delivery, 5‐minute Apgar score, and umbilical cord pH (all continuous). Variables specific to the neonatal period included the presence and severity of encephalopathy, convulsions in the first 72 hours, administration of antibiotics, hypoglycemia, hyperbilirubinemia, and sepsis (all binary or ordinal), as well as length of stay in hospital (continuous). Postneonatal brain injury was extracted (binary). Associated conditions were presence or absence of impairments in cognition, vision, hearing, and communication (binary). Interventions such as surgery or botulinum neurotoxin type A for tone, surgical tube feeding (gastrostomy or jejunostomy), or gavage feeds were documented as binary. Quantification of motor function was based on GMFCS and Manual Ability Classification System levels at older than 5 years (ordinal). Ambulation was considered a binary variable defined as GMFCS levels I to III. CP types were exclusive categories of spastic bilateral (diplegia, triplegia, quadriplegia), unilateral, ataxic, dyskinetic, and hypotonic.

There are eight previously established MRI patterns in the CCPR: normal, deep gray matter injury, white matter injury, white matter and cortical injury, near‐total brain injury, focal insult, malformation, or other (i.e. non‐specific).^20^ To enable independent groups for analysis only participants with a single MRI diagnosis were included. A subset of MRI reports was reviewed for evidence of stroke by a blinded pediatric stroke expert and categorized as definite, probable, or not stroke, and further categorized as arterial or venous when possible. Age at MRI was estimated by assigning each participant a birthday on the first day of their birth month and subtracting the MRI date from this approximate birth date (only birth month and year were available).

### Statistical methods

Descriptive statistics were used including frequency for categorical variables or medians with interquartile range (IQR) for continuous variables. Univariable comparison between the ‘undetectable’ and ‘detectable’ groups was performed by calculating the odds ratio (OR) with 95% CIs and *p*‐values. Fisher's exact test was used for variables with a zero‐count. All continuous variables such as age and weight were treated as such. For each participant, the CP risk calculator was applied (equation in Table S1) to generate a CP risk score from 0 to 1, and dichotomized at 0.3, on the basis of the previously suggested cut‐off score.^16^ The variables used in the risk calculator were not included in further analysis or tables, except for Tables S2 and S3.

Missing values for Table 1 were initially evaluated for randomness, then all the variables in Table 1 were used to perform multiple imputations using multivariable normal regression and at least 50 imputations or the fraction of missing information, whichever was larger. The output of the multiple imputation was used for multivariable logistic regression, adjusting coefficients and standard errors for the variability between imputations. Multivariable logistic regression was used to compare characteristics for variables with at least five participants per cell (adjusting for multiple imputations, when that technique was used). Multinomial regression was used to assess categorial outcomes with more than two categories (CP subtype and MRI pattern), and expressed as a relative risk ratio. For both multinomial and multivariable regression, all variables in the relevant table were used. Collinearity was evaluated for Table 1. Alpha was set at 0.01 owing to multiple comparisons. Participants included in the analysis were compared with those excluded owing to incomplete variables to mitigate bias (Table S2).

**TABLE 1:** Pregnancy, delivery, neonatal, postneonatal and impairment severity characteristics of children with CP with or without detectable perinatal risk factors.

		‘Undetectable’ CP risk: probability ≤0.3 without seizures or encephalopathy (*n* = 195)		‘Detectable’ CP risk: probability >0.3 or seizures or encephalopathy (*n* = 586)		Univariable		Multivariable (50 MI, FMI = 49)	
Variable type	Variables	*n* (%) or median (IQR)	Number, missing/total (%)	*n* (%) or median (IQR)	Number, missing/total (%)	OR (95% CI)	*p* (Fisher's exact test)	Odds ratio (95% CI)	*p*
Maternal/pregnancy	Trauma	22/194 (11.3%)	1/195 (0.5)	54/584 (9.3%)	2/586 (0.3)	1.3 (0.71–2.2)	0.4	1.3 (0.72–2.4)	0.37
	Alcohol	17/194 (8.8%)	1/195 (0.5)	83/581 (14.3%)	5/586 (0.8)	0.58 (0.31–1.0)	0.05	0.51 (0.28–0.93)	0.03
	Twin pregnancy	3/195 (1.5%)	0	22/585 (3.8%)	1/586 (0.2)	0.40 (0.08–1.4)	0.13	N/A	
	Any bleeding	39/193 (20.2%)	2/195 (1.0)	103/570 (18.1%)	16/586 (2.7)	1.2 (0.74–1.8)	0.51	1.2 (0.76–1.9)	0.43
	Intrauterine growth restriction	0/193 (0%)	2/195 (1.0)	39/583 (6.7%)	3/586 (0.5)	N/A	<0.001	N/A	
Delivery	Maternal fever	3/181 (1.7%)	14/195 (7.2)	56/527 (10.7%)	59/586 (10.0)	0.14 (0.03–0.45)	<0.001	N/A	
	Resuscitation at birth	7/192 (3.7%)	3/195 (2.0)	182/577 (31.5%)	9/586 (1.5)	0.08 (0.03–0.18)	<0.001	0.17 (0.07–0.41)	< 0.01
	Emergency Caesarean section	25/194 (12.9%)	1/195 (0.5)	172/586 (29.4%)	0	0.36 (0.22–0.57)	<0.001	0.62 (0.37–1.0)	0.07
Neonatal clinical	Cord pH	7.29 (7.24–7.334)	69/195 (35.4)	7.23 (7.1–7.31)	171/586 (29.1)	492.9 (62.3–3902)	<0.001	19.4 (2.0–184)	0.01
	Administration of antibiotics	4/194 (2.1%)	3/195 (2.0)	29/551 (5.3%)	35/586 (6.00)	0.38 (0.1–1.1)	0.06	N/A	
	Length of stay (days)	2 (2–3)	33/195 (17)	6 (2–16)	53/586 (9.0)	0.96 (0.93–0.98)	<0.001	1.0 (0.98–1.0)	0.53
	Hyperbilirubinemia	11/195 (5.6%)	0	56/586 (9.6%)	0	0.57 (0.26–1.1)	0.09	0.65 (0.31–1.3)	0.24
	Sepsis	2/191 (1.1%)	4/195 (2)	23/565 (4.1%)	21/586 (3.6)	0.25 (0.03–1.03)	0.04	N/A	
	Hypoglycemia	0/165 (0%)	30/195 (15.4)	3/513 (0.6%)	73/586 (12.4)	N/A	1.0	N/A	
	Seizures or encephalopathy	0	3/195 (2.0)	220/579 (38.0%)	7/586 (1.1)	N/A			
Postneonatal	Postneonatal brain injury	19/189 (10.1%)	6/195 (3.1)	52/575 (9.0%)	11/586 (1.9)	1.1 (0.61–2.0)	0.68	0.76 (0.41–1.4)	0.39
	Age at MRI (months)	18.6 (10.6–33)	28/195 (14.4)	12.7 (2.3–25.9)	96/586 (16.4)	1.0 (1.0–1.0)	0.015	1.0 (0.99–1.0)	0.9
	Surgery or botulinum neurotoxin type A	113/190 (59.5%)	5/195 (2.5)	335/564 (59.4%)	22/586 (3.8)	1.0 (0.7–1.4)	0.98	1.3 (0.86–1.8)	0.23
	Gastrostomy/jejunostomy	19/194 (9.8%)	1/195 (0.5)	77/581 (13.3%)	4/586 (0.7)	0.71 (0.4–1.2)	0.21	1.1 (0.48–2.6)	0.8
	Gavage feeds	12/195 (6.2%)	0	37/585 (6.3%)	1/586 (0.2)	0.97 (0.45–1.9)	0.93	1.6 (0.59–4.3)	0.36
Comorbid conditions	Cognitive impairment	60/154 (39.0%)	41/195 (21.0)	239/395 (60.5%)	191/586 (32.6)	0.41 (0.28–0.62)	<0.001	0.66 (0.36–1.2)	0.18
	Visual impairment	30/187 (16.0%)	8/195 (4.1)	115/557 (20.7%)	29/586 (5.0)	0.73 (0.46–1.2)	0.17	1.4 (0.74–2.6)	0.3
	Eyeglasses	28/173 (16.2%)	22/195 (11.3)	108/517 (20.9%)	69/586 (11.8)	0.73 (0.45–1.2)	0.18	0.79 (0.47–1.3)	0.36
	Auditory impairment	12/187 (6.4%)	8/195 (4.1)	54/558 (9.7%)	28/586 (4.8)	0.64 (0.3–1.3)	0.18	0.91 (0.43–1.9)	0.81
	Difficulties in communication	115/190 (60.5%)	5/195 (2.5)	413/567 (72.8%)	19/586 (3.2)	0.57 (0.40–0.82)	0.001	Words used	
Method of communication	Words	66/114 (57.9%)	81/195 (41.5)	154/409 (37.7%)	177/586 (30.2)	2.3 (1.5–3.6)	<0.001	1.4 (0.7–3.0)	0.32
	Words and non‐verbal system	17/114 (14.9%)	81/195 (41.5)	84/409 (20.5%)	177/586 (30.2)	0.67 (0.36–1.2)	0.18	Words used	
	Non‐verbal system only	9/114 (7.9%)	81/195 (41.5)	59/409 (14.4%)	177/586 (30.2)	0.51 (0.21–1.1)	0.07	Words used	
	No communication	19/114 (16.7%)	81/195 (41.5)	96/409 (23.5%)	177/586 (30.2)	0.65 (0.36–1.1)	0.12	Words used	
Degree of motor impairment	GMFCS severity at ≥5 years	I (I–II)	21/195 (10.8)	II (I–IV)	87/586 (14.8)	0.76 (0.67–0.86)	<0.001	0.84 (0.61–1.2)	0.3
	MACS severity at ≥5 years	II (I–III)	49/195 (25.1)	II (I–IV)	162/586 (27.6)	0.81 (0.71–0.92)	0.001	1.1 (0.79–1.5)	0.61
	Non‐ambulatory CP	27/174 (15.5%)	21/195 (10.8)	151/499 (30.3%)	87/586 (14.8)	0.42 (0.26–0.67)	<0.001	Used GMFCS	

Abbreviations: CI, confidence interval; CP, cerebral palsy; FMI, fraction of missing information; GMFCS, Gross Motor Function Classification System; IQR, interquartile range; MACS, Manual Ability Classification System; MI, multiple imputations; MRI, magnetic resonance imaging; N/A, not applicable owing to low numbers or substitute variable used.

The data from the CCPR were analyzed from June 2023 to August 2023 with IBM SPSS (IBM Corp., Armonk, NY, USA) and STATA version 15 (StatCorp, College Station, TX, USA) and then re‐analyzed May 2024 with STATA version 18 in response to reviewers' suggestions.

## RESULTS

**FIGURE 1:**
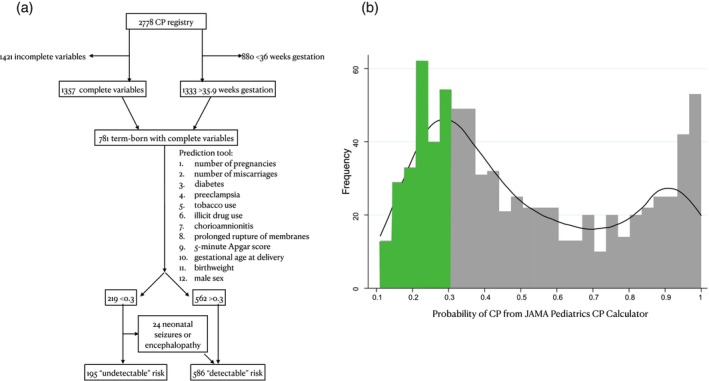
Study participants from the Canadian Cerebral Palsy Registry. (a) Flowchart demonstrating selection of participants, with variables included in the risk stratification tool, and the movement of 24 participants to the ‘detectable’ group owing to neonatal symptoms (encephalopathy and/or seizures). (b) Histogram of output from the risk stratification tool; participants with cerebral palsy (CP) falling below the 0.3 probability threshold are highlighted in green.

There were 2778 registry participants screened; 1333 (48%) were of at least 37 weeks gestational age at birth and 781 (58.6%) had complete variables for the CP risk calculator (Figure 1a). Characteristics of those included and excluded owing to missing calculator variables were compared and found to be comparable (Table S2). There were 71 out of 764 (9.3%) with postneonatal CP. After applying the CP risk calculator, 219 out of 781 (28.0%) participants had a score of less than or equal to 0.3, and 562 out of 781 (72.0%) had a score greater than 0.3 (Figure 1a). The distribution of scores was bimodal, with peaks around 0.25 and 0.9 (Figure 1b). Presence or absence of neonatal encephalopathy was documented in 772 out of 781 (98.9%), and was present in 204 out of 772 (26.4%), of whom 5 out of 204 (2.5%) were mild, 135 out of 204 (66.2%) were moderate, and 64 out of 772 (31.3%) were severe. Seizures in the first 72 hours after delivery were present in 177 out of 772 (22.9%). Either seizures and/or neonatal encephalopathy were documented as present in 220 out of 771 (28.5%). There were 24 out of 219 (11.0%) participants in the group with a score less than or equal to 0.3 who had encephalopathy or seizures and were thus moved to the ‘detectable’ group.

The final study population was 195 out of 781 (25.0%) participants with ‘undetectable’ neonatal CP risk (calculator score ≤0.3 and no seizures or encephalopathy), and 586 out of 781 (75%) with ‘detectable’ CP risk (calculator score >0.3, or neonatal seizures or encephalopathy) (Figure 1). The characteristics of the groups are shown in Table 1. The proportion of males was 93 out of 195 (47.7%) in the ‘undetectable’ group and 354 out of 586 (60.4%) in the ‘detectable’ group (OR 0.60, 95% CI 0.43–0.84); sex is one of the 12 variables on which the CP risk calculator is based. The results for the additional 11 variables of the CP risk calculator are shown in Table S3. The provinces of birth for each group are available in Table S4.

On univariable analysis of pregnancy, delivery, and neonatal variables, participants with ‘undetectable’ CP risk had fewer instances of: maternal fever at delivery (OR 0.14, 95% CI 0.03–0.45), emergency Caesarean sections (OR 0.36, 95% CI 0.022–0.57), resuscitation (OR 0.08, 95% CI 0.03–0.18), days in hospital (OR 0.96, 95% CI 0.93–0.98); and they had higher cord pH (OR 492.9, 95% CI 62.3–3902). The ‘undetectable’ group tended to have less hyperbilirubinemia, sepsis, and hypoglycemia; however, the differences were not significant. There were no participants in the ‘undetectable’ group with intrauterine growth restriction, but 39 out of 583 (6.7%) had intrauterine growth restriction in the ‘detectable’ group (*p* < 0.001).

There was no difference in the proportion with postneonatal CP (10.1% vs 9.0%, OR 1.1, 95% CI 0.61–2.0). Both cognitive and communication impairment were less common in those with undetectable neonatal CP risk (OR 0.41, 95% CI 0.28–0.62, and OR 0.57, 95% CI 0.4–0.82), and they were more likely to communicate with words (OR 2.3, 95% CI 1.5–3.6). GMFCS levels at 5 years or more were lower (higher motor function) in those with undetectable neonatal CP risk (OR 0.76, 95% CI 0.67–0.86), as were severity scored by the Manual Ability Classification System (OR 0.81, 95% CI 0.71–0.92) and the odds of being non‐ambulatory (OR 0.42, 95% CI 0.26–0.67).

With multivariable analysis using 50 multiple imputations, the only variables that remained significantly different between groups were resuscitation at birth (OR 0.17, 95% CI 0.07–0.41) and cord pH (OR 19.4, 95% CI 2.0–184) (Table 1), with a mean variance inflation factor on collinearity testing of 1.97, and no variance inflation factor values greater than 7.0.

### Missing values

Data were complete for the 12 variables on which the CP risk calculator was based. For the remaining variables in Table 1, data were complete for 61% of participants, and the most commonly missing variable was umbilical cord/arterial pH, missing in 28% of participants, followed by maternal fever (missing in 7%), then maternal fever and pH (2%). Missing data were handled in Table 1 with 50 multiple imputations (fraction of missing information 49%) using all the variables in Table 1 to ensure sufficient variables predictive of missing values. Missing values are provided in Table 1 for each variable.

**FIGURE 2:**
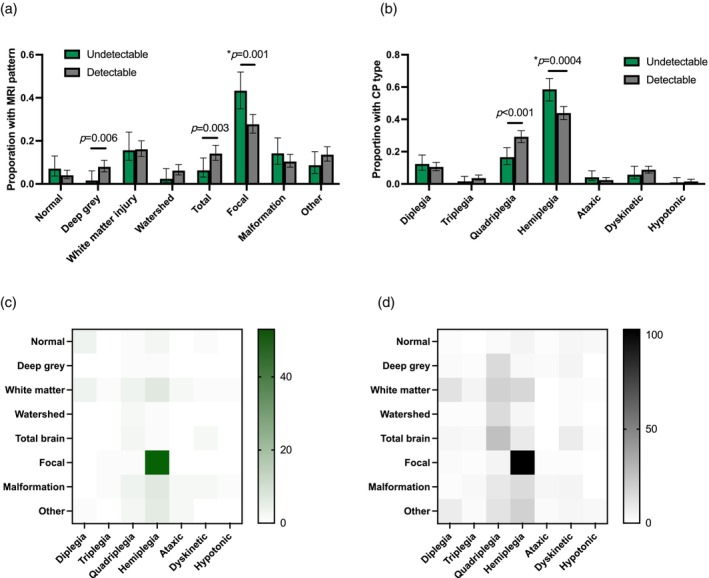
Magnetic resonance imaging (MRI) and cerebral palsy (CP) characteristics for participants with CP with and without perinatal risk factors and/or neonatal encephalopathy or seizures. (a) MRI patterns. (b) CP types. (c) Heatmap of MRI versus CP type for those without detectable CP risk. (d) Heatmap of MRI versus CP type for those with detectable CP risk. *Denotes univariable *p*‐value when variable was used as the reference for multinomial analysis. Green, ‘undetectable’ CP risk factors; gray, ‘detectable’ risk factors.

MRI categorization was available for 127 out of 195 (65.1%) and 404 out of 586 (68.3%) for the ‘undetectable’ and ‘detectable’ groups respectively (Table 2). There were five participants' MRIs excluded for dual diagnosis. On univariable analysis, focal injury was more common (OR 2.0, 95% CI 1.3–3.1) in the ‘undetectable’ group (Figure 2a). On multinomial analysis using focal injury as the baseline, there was a lower relative risk of deep gray matter injury (relative risk ratio 0.13, 95% CI 0.03–0.55) and total brain injury (relative risk ratio 0.29, 95% 0.13–0.64) for those with undetectable neonatal risk (Table 2). A subset of MRI reports (252 out of 531; 47%) were evaluated for stroke. Probable or definite stroke was found in 63 out of 76 (82.9%) of the ‘undetectable’ group and 122 out of 176 (69.3%) of the ‘detectable’ group (OR 2.2, 95% CI 1.1–4.6). The overall estimated incidence of stroke in those with MRI categorization was 63 out of 127 (32.3%) for the ‘undetectable’ group and 122 out of 404 (30.2%) for the ‘detectable’ group. There was more venous stroke in the ‘undetectable’ group (34 out of 61; 55.7% vs 40 out of 113; 35.4%; OR 2.3, 95% CI 1.2–4.6). The median age at MRI was 18.6 months (IQR 10.6–33) compared with 12.7 months (IQR 2.3–25.9) for the ‘undetectable’ vs ‘detectable’ groups (*p* = 0.001).

**TABLE 2:** Imaging characteristics of those with and without detectable neonatal risk factors or symptoms.

		Undetectable (*n* = 195)		Detectable (*n* = 586)		Univariable		Multinomial	
		*n*	Missing (%)	*n*	Missing (%)	Odds ratio (95% CI)	*p*	RRR (95% CI)	*p*
	Age at MRI (median months, IQR)	18.6 (10.6–33)	28/195 (14.6)	12.7 (2.3–29.5)	96/586 (16.3)	N/A	0.001		
Expert MRI diagnosis	Normal	9/127 (7.1%)	68/195 (34.9)	16/404 (4.0)	182/586 (31.1)	1.9 (0.7–4.6)	0.15	1.2 (0.48–2.8)	0.75
	Deep gray matter injury	2/127 (1.6%)	68/195 (34.9)	32/404 (7.9%)	182/586 (31.1)	0.19 (0.02–0.75)	0.011	0.13 (0.03–0.55)	0.006
	White matter injury	21/127 (16.5%)	68/195 (34.9)	65/404 (16.1%)	182/586 (31.1)	1.0 (0.57–1.8)	0.9	0.66 (0.37–1.2)	0.17
	Watershed	3/127 (2.4%)	68/195 (34.9)	25/404 (6.2%)	182/586 (31.1)	0.37 (0.07–1.2)	0.09	0.26 (0.07–0.85)	0.027
	Near‐total brain injury	8/127 (6.3%)	68/195 (34.9)	57/404 (14.1%)	182/586 (31.1)	0.41 (0.16–0.90)	0.019	0.29 (0.13–0.64)	0.003
	Focal injury	55/127 (43.3%)	68/195 (34.9)	112/404 (27.7%)	182/586 (31.1)	2.0 (1.3–3.1)	0.001	Reference	
	Malformation	18/127 (14.2%)	68/195 (34.9)	42/404 (10.4%)	182/586 (31.1)	1.4 (0.74–2.6)	0.24	0.88 (0.47–1.7)	0.7
	Other	11/127 (8.7%)	68/195 (34.9)	55/404 (13.6%)	182/586 (31.1)	0.60 (0.27–1.2)	0.14	0.47 (0.24–0.93)	0.03
Stroke evaluation	Stroke review done	76/127 (59.8%)	51/127 (40.1)	177/404 (43.8%)	227/404 (56.2)				
	Stroke probable or definite	63/76 (82.9%)	51/127 (40.1)	122/176 (69.3%)	227/404 (56.2)	2.2 (1.1–4.6)	0.025		
	Arterial stroke	27/61 (44.3%)	51/127 (40.1)	73/113 (64.6%)	227/404 (56.2)	0.44 (0.22–0.86)	0.01		
	Venous stroke	34/61 (55.7%)	51/127 (40.1)	40/113 (35.4%)	227/404 (56.2)	2.3 (1.2–4.6)	0.01		

Abbreviations: CI, confidence interval; IQR, interquartile range; MRI, magnetic resonance imaging; RRR, relative risk ratio.

Subtypes of CP are given in Table 3 and Figure 2b and d. Those in the ‘undetectable’ group had more unilateral spastic CP (OR 1.8, 95% CI 1.3–2.6) and less bilateral (quadriplegia) (OR 0.48, 95% CI 0.31–0.74), which persisted in multinomial analysis. Figure 2c,d shows heatmaps of MRI pattern and CP subtype to illustrate the different phenotypes between groups.

**TABLE 3:** Types of cerebral palsy.

	Undetectable (*n* = 195)		Detectable (*n* = 586)		Univariable		Multinomial	
	Proportion (%)	Number, missing/total (%)	Proportion (%)	Number, missing/total (%)	Odds ratio (95% CI)	*p* (Fisher's exact test)	RRR (95% CI)	*p*
Spastic bilateral (diplegia)	24/193 (12.4)	2/195 (1)	61/579 (10.5)	7/586 (1)	1.2 (0.69–2.0)	0.46	0.88 (0.52–1.5)	0.64
Spastic bilateral (triplegia)	3/193 (1.6)	2/195 (1)	21/579 (3.6)	7/586 (1)	0.42 (0.08–1.4)	0.15	0.32 (0.09–1.1)	0.07
Spastic bilateral (quadriplegia)	32/193 (16.6)	2/195 (1)	169/579 (29.2)	7/586 (1)	0.48 (0.31–0.74)	0.001	0.43 (0.27–0.66)	<0.001
Unilateral spastic	113/193 (58.6)	2/195 (1)	254/579 (43.9)	7/586 (1)	1.8 (1.3–2.6)	<0.001	Reference	
Ataxic	8/193 (4.2)	2/195 (1)	14/579 (2.4)	7/586 (1)	1.7 (0.62–4.5)	0.21	1.3 (0.52–3.2)	0.58
Dyskinetic	11/193 (5.7)	2/195 (1)	51/579 (8.8)	7/586 (1)	0.63 (0.29–1.2)	0.17	0.49 (0.24–0.96)	0.04
Hypotonia	2/193 (1.0)	2/195 (1)	9/579 (1.5)	7/586 (1)	0.66 (0.07–3.3)	0.6	0.50 (0.11–2.4)	0.38

Abbreviations: CI, confidence interval; RRR, relative risk ratio.

## DISCUSSION

We found that participants in the CCPR born at term with ‘undetectable’ neonatal risk were more likely to have unilateral CP and focal injury on MRI, particularly venous stroke, than those with perinatal risk factors and/or symptoms, and they had a lower degree of impairments as measured by the GMFCS and were more likely to communicate with words. Contrary to our hypothesis, the ‘undetectable’ group did not have more postneonatal causes of CP.

The lack of neonatal symptoms and risk factors in the ‘undetectable’ group may reflect an early, antenatal timing of disruption to the central nervous system leading to CP. In this study, 28% of participants had encephalopathy or seizures, which is similar to other studies.^21^, ^22^ There has been speculation that the absence of documented neonatal symptoms in some children with CP represents encephalopathy that has been ‘missed’ and could have benefited from interventions such as therapeutic hypothermia. Contrary to that hypothesis, the HEAL Trial has shown that subacute injuries (>7–10 days, on the basis of the absence of diffusion restriction)^23^ can be associated with ‘mild’ encephalopathy, which suggests previous injury to the brain allowing for some resolution of encephalopathy before delivery.^24^ This conclusion is further supported by high rates of MRI abnormalities in infants with mild encephalopathy,^25^ and a recent study from the CCPR that found that 17% of participants with MRI patterns of hypoxia–ischemia did not have documented neonatal encephalopathy (nor postneonatal injury).^26^ Postneonatal brain injury in the CCPR includes infection, trauma, medical intervention, stroke, hypoxia (near‐drowning or near‐sudden infant death syndrome), and miscellaneous,^7^, ^8^ and contrary to our hypothesis was found in approximately 9% of participants in both groups, which is similar to other studies reporting 6% to 8%.^8^, ^9^, ^10^, ^27^ Genetic conditions are another important cause of CP that may be asymptomatic at birth, which are considered to account for 7% to 30% of CP.^28^, ^29^, ^30^, ^31^, ^32^ The characteristics of children with genetic causes of CP include those with intellectual disability, born at term, with a normal MRI, or non‐stroke MRI abnormalities.^33^, ^34^ Children without detectable risk factors have a higher prevalence of genetic causes for their CP.^35^

Focal injury including probable or definite stroke was diagnosed more in the ‘undetectable’ group, and they had more venous stroke than the ‘detectable’ group. Venous stroke accounts for 60% of perinatal stroke that is silent at birth, the most common being periventricular infarction due to antenatal germinal matrix hemorrhage,^36^ which results in CP in approximately three‐quarters of cases.^37^ Malformations were not more common in the ‘undetectable’ group, which was somewhat surprising given that early antenatal injury or genetic conditions causing malformations could lack detectable perinatal risk factors.

Supporting the finding of more stroke in the ‘undetectable’ group was the prevalence of unilateral CP, higher rates of ambulation, and better hand function. In addition to milder physical impairments, the group lacking detectable risk factors had a lower prevalence of cognitive and communication impairments, and were more likely to communicate with words. This is consistent with other studies finding more severe CP in term‐born children with neonatal symptoms.^38^, ^39^

Because many of the participants with ‘undetectable’ neonatal risk had predominantly unilateral CP, screening efforts could be focused on supporting primary care providers to detect early signs of hemiparesis. The Early Detection and Intervention Toolkit for CP^40^ is an ongoing Canadian knowledge translation study to support primary care providers in identifying clues on physical examination that should prompt referral to a specialist for evaluation of CP. The Hammersmith Infant Neurological Examination is a clinical examination that can easily be performed and has high sensitivity and specificity for unilateral CP.^41^, ^42^, ^43^ An important message is that a hand preference before the age of 1 year is pathological.^44^

This study has several limitations. It was a cross‐sectional study where data collection was limited by availability in the chart, such as documentation of encephalopathy. We did not have access to MRI, only textual MRI reports that were categorized according to standard criteria as previously described.^17^, ^38^, ^45^ Although multiple imputation was used to address missing values and reduce bias, caution should be used when interpreting the results of variables with a high proportion of missing values. Only a subset of the CCPR with variables complete for the CP risk calculator was used; however, this subset was comparable to the other term‐born registry participants for the variables in Table 1 (Table S2). Although a standardized, validated CP risk calculator was used to help categorize detectable versus undetectable CP, the results were limited by the performance of the model in the calculator.^16^ Since this is a multi‐provincial registry, the results are expected to be applicable to children with CP in settings with similar resources.

## CONCLUSIONS

In this national registry from a high‐resource setting, children with CP born at term lacking detectable perinatal CP risk factors were more likely to be female, have an uneventful pregnancy and delivery, have more focal injury (including perinatal stroke), have predominantly hemiparetic/unilateral CP, be ambulatory, and communicate with words more than children with CP who had detectable risk factors. They were not more likely to have experienced a postneonatal brain injury, and we propose the etiology of their CP may have been an initially asymptomatic perinatal brain injury (such as antenatal germinal matrix hemorrhage with venous infarct). These findings are expected to be generalizable to other populations with similar perinatal care and resources. Understanding this phenotype, which encompasses a quarter of children with CP in our registry, helps explain the limitations of CP screening tools based on perinatal risk factors, and highlights the importance of supporting primary care providers in recognizing early signs of CP such as subtle hemiparesis, and initiating prompt referral for further evaluation and treatment.

## FUNDING INFORMATION

This study was not specifically funded; however, the Canadian Cerebral Palsy Registry has received funds from Kids Brain Health Network (formerly known as NeuroDevNet), the Public Health Agency of Canada, and the Harvey Guyda Chair in Pediatrics Fund of the Montreal Children's Hospital Foundation. The work of MJD and KS is funded by the Alberta Children's Hospital Research Institute.

## CONFLICT OF INTEREST STATEMENT

The authors have stated that they had no interests that might be perceived as posing a conflict or bias.

## Supporting information


**Table S1:** Variable definitions.

**Table S2:** Comparison of participants included and excluded from the study.

**Table S3:** Results of 12 variables on which the CP risk calculator is based.

**Table S4:** Province of birth for participants.

## Data Availability

The data that support the findings of this study are available from the corresponding author, MJD, upon reasonable request.
