# Supplementary material for: Cerebral palsy characteristics in term‐born children with and without detectable perinatal risk factors: A cross‐sectional study
Source: Dev Med Child Neurol. 2024 Oct 15;67(4):475–85. doi: 10.1111/dmcn.16111 (PMC11875524; doi:10.1111/dmcn.16111)
Supplement: Supplementary file 1 — Table S1: Variable definitions. [file DMCN-67-475-s001.docx]

Etable 1: variable definitions

| Variable | Coding type | Definition |
| --- | --- | --- |
| **Trauma** | Binary | Physical trauma experienced during the pregnancy, further characterized as direct trauma to abdomen, indirect trauma to abdomen, motor vehicle accident, abdominal surgery, or other |
| **Alcohol use** | binary | Coded as yes for any exposure in pregnancy |
| **Twin pregnancy** | Binary | Two fetuses at any time during the pregnancy |
| **Vaginal bleeding** | Binary | Self reported or documented vaginal bleeding in any trimester of pregnancy |
| **Intrauterine growth restriction** | Binary | Documentation from the antenatal record of concern for IUGR, defined as fetal length less than two standard deviations (or third percentile) below the mean for gestational age |
| **Maternal fever in labor** | binary | Temperature measured >38.0°C during labor |
| **Resuscitation** | Binary | Documentation in the chart of Intubation, cardiac massage, epinephrine, IV bolus |
| **Emergency Caesarian section** | binary | Caesarian section that was not scheduled |
| **pH** | continuous | Arterial umbilical artery pH or arterial pH less than 1 hour after delivery |
| **Administration of Antibiotics** | binary | Documented administration of any antibiotic in the medical chart |
| **Length of stay in hospital** | continuous | Duration of initial hospitalization at any level of care (including NICU) as documented in the discharge summary |
| **Hyperbilirubinemia** | binary | Blood bilirubin values greater than cut-off for age |
| **Sepsis** | binary | Bacteremia with evidence of inflammation and/or end organ dysfunction |
| **Hypoglycemia** | binary | blood glucose level <3 mmol/L |
| **Encephalopathy** | binary | Depressed level of consciousness, variably associated with respiratory depression, abnormalities of tone and power, disturbances of cranial nerve function (including impaired feeding), and often seizures  Source: Volpe, J. J. Neonatal encephalopathy: An inadequate term for hypoxic–ischemic encephalopathy. *Ann Neurol* **72**, 156–166 (2012).  Determined by chart review by research assistant |
| **Seizure** | binary | Positive if clinical or electrographic seizure recorded in first 72 hours of life |
| **Post-neonatal brain injury** | Binary | Brain injury believed to be causative for CP between 28 days post delivery and 2^nd^ birthday, further categorized as: perisurgical cerebrovascular accident, other cerebrovascular accident, post drowning anoxia, post SIDS anoxia, post convulsion anoxia, other anoxia, hydrocephalus, bacterial infection, viral infection, other infection, coagulation problem, immunological problem, head injury, shaken baby syndrome, other |
| **Age at MRI** | Continuous | Estimated by assigning the participant a birthday on the first day of their birth month/year, and subtracting this from the date of the MRI. |
| **Surgery or botox** | binary | History of surgery for tone such as tendon release, or botox injection at any time |
| Gastrostomy /Jejunostomy | binary | permanent artificial tube to administer the bulk of enteral nutrition |
| **Gavage feeding** | binary | temporary artificial tube to administer the bulk of enteral nutrition |
| **Cognitive impairment** | binary | age-appropriate standardized testing undertaken by the local center indicating intellectual disability |
| **Visual impairment** | binary | cortical blindness, diagnosed by an ophthalmologist |
| **Require eyeglasses** | binary | Glasses prescribed by a qualified optometrist |
| **Auditory impairment** | binary | 70 dB or greater hearing loss on audiometric testing |
| **Difficulty with Communication** | binary | nonverbal, defined as the absence of specific words or recognizable vocabulary in the child’s maternal language |
| **GMFCS** | Ordinal | Gross Motor Function Classification System:  **GMFCS Level I**  Children walk at home, school, outdoors and in the community. They can climb stairs without the use of a railing. Children perform gross motor skills such as running and jumping, but speed, balance and coordination are limited.  **GMFCS Level II**  Children walk in most settings and climb stairs holding onto a railing. They may experience difficulty walking long distances and balancing on uneven terrain, inclines, in crowded areas or confined spaces. Children may walk with physical assistance, a handheld mobility device or used wheeled mobility over long distances. Children have only minimal ability to perform gross motor skills such as running and jumping.  **GMFCS Level III**  Children walk using a hand-held mobility device in most indoor settings. They may climb stairs holding onto a railing with supervision or assistance. Children use wheeled mobility when travelling long distances and may self-propel for shorter distances.  **GMFCS Level IV**  Children use methods of mobility that require physical assistance or powered mobility in most settings. They may walk for short distances at home with physical assistance or use powered mobility or a body support walker when positioned. At school, outdoors and in the community children are transported in a manual wheelchair or use powered mobility.  **GMFCS Level V**  Children are transported in a manual wheelchair in all settings. Children are limited in their ability to maintain antigravity head and trunk postures and control leg and arm movements. |
| **MACs** | Ordinal | Manual Ability Classification System (MACS)   1. **Handles objects easily and successfully**. At most, limitations in the ease of performing manual tasks requiring speedand accuracy. However, any limitations in manual abilities do not restrict independence in daily activities. 2. **Handles most objects but with somewhat reduced quality and/or speed of achievement** – Certain activities may be avoided or be achieved with some difficulty; alternative ways of performance might be used but manual abilities do not usually restrict independence in daily activities. 3. **Handles objects with difficulty; needs help to prepare and/or modify activities** – The performance is slow and achieved with limited success regarding quality and quantity. Activities are performed independently if they have been set up or adapted. 4. **Handles a limited selection of easily managed objects in adapted situations** – Performs parts of activities with effort and with limited success. Requires continuous support and assistance and/or adapted equipment, for even partial achievement of the activity. 5. **Does not handle objects and has severely limited ability to perform even simple actions.** – Requires total assistance. |
| **Non-ambulatory** | binary | GMFCS IV-V |
| **Variables in the CP risk Calculator** | | |
| **Equation:** Log(pCP/1-pCP) = number of pregnancies(0.31) + number of miscarriages(-0.29) + tobacco(0.82) + drugs(2.34) + diabetes(0.75) + pre-eclampsia(1.39) + chorioamnionitis(2.7) + prolonged rupture of membranes(-0.69) + 5-minute Apgar score(-0.44) + gestational age at birth(-0.11) + male sex(0.21) + birthweight in kg(-2.2) + birthweight in kg^2^(0.27) + 10.9 The equation was applied to each participant in the study and an odds and probability of CP determined (by exponentiating and then odds/(1+odds), respectively). | | |
| **Number of pregnancies** | continuous | Number of mother's total pregnancies |
| **Number of miscarriages** | continuous | Number of miscarriages: number of pregnancies that spontaneously aborted prior to 20 weeks gestation |
| **Diabetes** | binary | Gestational Diabetes diagnosed as recommended by the Society of Obstetricians and Gynaecologist of Canada:  **1**      The “preferred screening and diagnostic 2-step” approach for gestational diabetes mellitus from Diabetes Canada's 2018 guidelines is endorsed. All pregnant women should be offered screening between 24-28 weeks using a standardized non-fasting 50-g glucose challenge screening test (GCT) with plasma glucose (PG) measured 1 hour later (III-B).  **1.1**  If the value is <7.8 mmol/L, no further testing is required.  **1.2**  If the value of the GCT is 7.8–11.0, a 2-hour 75-g oral glucose tolerance test with fasting PG (FPG), 1-hour PG, 2-hour PG should be performed. Gestational diabetes mellitus is diagnosed if one value is met or exceeded:  **1.2.1**       FPG ≥5.3 mmol/L  **1.2.2**       1-h PG ≥10.6 mmol/L  **1.2.3**       2-h PG ≥9.0 mmol/L  **1.3**  If the value of the GCT is ≥11.1 mmol/L, gestational diabetes mellitus is diagnosed  **2**      The “alternative 1-step diagnostic” approach from Diabetes Canada's 2018 guidelines is acceptable. In this strategy pregnant women should be offered testing between 24-28 weeks using a standardized 2-hour 75-g oral glucose tolerance test with fasting plasma glucose (FPG), 1-hour plasma glucose (PG), 2-hour PG (III-B). Gestational diabetes mellitus is diagnosed if 1 value is met or exceeded:  **2.1.1**       FPG ≥5.1 mmol/L  **2.1.2**       1-h PG ≥10.0 mmol/L  **2.1.3**       2-h PG ≥8.5 mmol/L  *Source: Guideline No. 393-Diabetes in Pregnancy Berger, H., Gagnon, R, Sermer, M. et al. Journal of Obstetrics and Gynaecology Canada , Volume 41, Issue 12, 1814 - 1825.e1* [*https://www.jogc.com/article/S1701-2163(19)30298-1/fulltext*](https://www.jogc.com/article/S1701-2163(19)30298-1/fulltext) |
| **Pre-eclampsia** | binary | Diagnosed as guided by the Society of Obstetricians and Gynecologists of Canada:  Preeclampsia is defined as gestational hypertension with one or more of the following:  ● new proteinuria, or  ● one or more adverse conditions,* or ● one or more severe complications.* Severe preeclampsia is defined as preeclampsia with one or more severe complications.    The diagnosis of hypertension should be based on office or in-hospital blood pressure measurements. (II-B) Hypertension in pregnancy should be defined as an office (or in-hospital) systolic blood pressure ≥ 140 mmHg and/or diastolic blood pressure ≥ 90 mmHg, based on the average of at least 2 measurements, taken at least 15 minutes apart, using the same arm. (II-2B)  All pregnant women should be assessed for proteinuria. (II-2B) Urinary dipstick testing (by visual or automated testing) may be used for screening for proteinuria when the suspicion of preeclampsia is low. (II-2B)  Significant proteinuria should be defined as ≥ 0.3 g/d in a complete 24-hour urine collection or ≥ 30 mg/mmol urinary creatinine in a spot (random) urine sample. (II-2B)  Significant proteinuria should be suspected when urinary dipstick proteinuria is ≥ 1+. (II-2A)  More definitive testing for proteinuria (by urinary protein:creatinine ratio or 24-hour urine collection) is encouraged when there is a suspicion of preeclampsia, including: ≥ 1+ dipstick proteinuria in women with hypertension and rising blood pressure and in women with normal blood pressure, but symptoms or signs suggestive of preeclampsia. (II-2A)  *Source: Diagnosis, Evaluation, and Management of the Hypertensive Disorders of Pregnancy: Executive Summary Magee, Laura et al. Journal of Obstetrics and Gynaecology Canada , Volume 36, Issue 5, 416 - 438*  <https://www.jogc.com/article/S1701-2163(15)30588-0/fulltext> |
| **Chorio-amnionitis** | binary | Counted as present if clinically diagnosed according to American College of Obstetrics and Gynecology guidelines, acknowledging practice variability center-to-center:  Source: 1.Charpentier, C. *et al.* A Survey on Variation in Diagnosis and Treatment of Chorioamnionitis in Tertiary Centres in Canada. *J Obstetrics Gynaecol Can* **44**, 28–33 (2022). |
| **Tobacco use** | binary | Coded as yes for any exposure in pregnancy |
| **Drug use** | binary | Coded as yes for any exposure in pregnancy |
| **Prolonged rupture of membranes(>18hrs)** | binary | Rupture of membranes greater than 18 hours |
| **5-minute Apgar Score** | continuous | Apgar score at five minutes post-delivery from the birth record |
| **Male sex** | binary | Infant born with external male genitalia |
| **Birth weight (kg)** | continuous | First weight measured at birth in kilograms |
| **Birth weight (kg^2^)** | continuous | First weight measured at birth in kilograms x weight measure at birth in kilograms |
| **Gestational age (weeks)** | continuous | Gestational age as measured in weeks post-menstrual age, or weeks post conceptual age in the case of in vitro fertilization |
