# Supplementary material for: Cerebral palsy characteristics in term‐born children with and without detectable perinatal risk factors: A cross‐sectional study
Source: Dev Med Child Neurol. 2024 Oct 15;67(4):475–85. doi: 10.1111/dmcn.16111 (PMC11875524; doi:10.1111/dmcn.16111)
Supplement: Supplementary file 2 — Table S2: Comparison of participants included and excluded from the study. [file DMCN-67-475-s004.docx]

Etable 2: comparison of participants included and excluded from the study

|  |  | **+ prediction tool (included in present study, n=718)** | | | | | **No prediction tool (excluded from present study, n=1088)** | | | | | **OR (95% CI)** | **P-value** | **Multivariable (50 MI, n=1826)** | **P-value** |
| --- | --- | --- | --- | --- | --- | --- | --- | --- | --- | --- | --- | --- | --- | --- | --- |
|  |  | n | Proportion | Median (IQR) | Missing | % missing | n | Proportion | Median (IQR) | Missing | % missing |  |  |  |  |
| **Maternal/Pregnancy** | *No. of pregnancies | 781 |  | 2 (1-3) | 0 | 0.0% | 575 |  | 2 (1-3) | 513 | 47.2% | 1.1 (1.0-1.2) | 0.032 | 1.1 (0.99-1.2) | 0.07 |
|  | Trauma | 778 | 9.7% |  | 3 | 0.4% | 561 | 8.0% |  | 527 | 48.4% | 0.84 (0.56-1.3) | 0.39 | 0.94 (0.65-1.4) | 0.76 |
|  | *No. of Miscarriages | 781 |  | 0 (0-1) | 0 | 0.0% | 559 |  | 0 (0-1) | 529 | 48.6% | 1.02 (0.89-1.2) | 0.89 | 0.91 (0.75-1.1) | 0.32 |
|  | *Gestational Diabetes | 781 | 10% |  | 0 | 0.0% | 556 | 6.7% |  | 532 | 48.9% | 0.65 (0.42-0.97) | 0.047 | 0.76 (0.51-1.1) | 0.19 |
|  | *Preeclampsia | 781 | 4.4% |  | 0 | 0.0% | 454 | 3.8% |  | 634 | 58.3% | 0.92 (0.47-1.7) | 0.78 | 0.92 (0.5-1.7) | 0.79 |
|  | *Chorioamnionitis | 781 | 10.2% |  | 0 | 0.0% | 81 | 5.8% |  | 1007 | 92.6% | 0.45 (0.11-1.2) | 0.12 | 0.93 (0.58-1.5) | 0.74 |
|  | *Tobacco | 781 | 16.8% |  | 0 | 0.0% | 511 | 15.8% |  | 577 | 53.0% | 0.93 (0.68-1.3) | 0.70 | 1.0 (0.73-1.4) | 0.86 |
|  | *Drugs | 781 | 3.8% |  | 0 | 0.0% | 578 | 3.5% |  | 510 | 46.9% | 0.79 (0.41-1.5) | 0.43 | 0.93 (0.49-1.7) | 0.82 |
|  | Alcohol | 775 | 12.9% |  | 6 | 0.8% | 514 | 10.2% |  | 574 | 52.8% | 0.77 (0.53-1.1) | 0.11 | 0.80 (0.56-1.1) | 0.21 |
|  | Twin Pregnancy | 780 | 3.2% |  | 1 | 0.1% | 672 | 13.8% |  | 416 | 38.2% | 6.4 (3.8-11.4) | **<0.0001** | **2.5 (1.4-4.5)** | **0.003** |
| **Bleeding during pregnancy** | 1st Trimester | 764 | 13.0% |  | 17 | 2.2% | 551 | 11.8% |  | 537 | 49.4% | 0.90 (0.64-1.3) | 0.52 | 1.0 (0.75-1.4) | 0.94 |
|  | 2nd Trimester | 762 | 4.3% |  | 19 | 2.4% | 550 | 3.3% |  | 538 | 49.4% | 0.75 (0.40-1.4) | 0.33 |  |  |
|  | 3rd Trimester | 763 | 4.1% |  | 18 | 2.3% | 546 | 3.9% |  | 542 | 49.8% | 0.95 (.52-1.7) | 0.84 |  |  |
| **Delivery** | Maternal Fever | 708 | 8.3% |  | 73 | 9.3% | 392 | 8.3% |  | 696 | 64.0% | 1.0 (0.62-1.6) | 0.98 | 1.1 (0.64-1.8) | 0.75 |
|  | Resuscitation at Birth | 769 | 24.6% |  | 12 | 1.5% | 512 | 26.0% |  | 576 | 52.9% | 1.1 (0.83-1.4) | 0.61 | 1.3 (0.81-2.0) | 0.33 |
|  | Emergency C-Section | 780 | 25.3% |  | 1 | 0.1% | 538 | 24.6% |  | 550 | 50.6% | 0.96 (0.74-1.2) | 0.88 | 1.0 (0.76-1.3) | 1.0 |
|  | *Prolonged Rupture of Membranes | 781 | 6.4% |  | 0 | 0.0% | 448 | 10.3% |  | 640 | 58.8% | 1.8 (1.2-2.8) | **0.009** | **3.0 (2.2-4.1)** | **<0.0001** |
|  | *Apgar at 5 min | 781 |  | 9 (7-9) | 0 | 0.0% | 436 |  | 9 (7-9) | 652 | 59.9% | 0.99 (0.94-1.04) | 0.77 | 1.0 (0.95-1.1) | 0.49 |
|  | Cord pH | 541 |  | 7.25 (7.14-7.31) | 240 | 30.7% | 318 |  | 7.24 (7.11-7.3) | 770 | 70.8% | 0.68 (0.31-1.5) | 0.34 | 0.77 (0.31-1.9) | 0.64 |
| **Infant** | *Male | 781 | 57.3% |  | 0 | 0.0% | 1064 | 56.5% |  | 24 | 2.2% | 0.97 (0.8-1.2) | 0.76 | 0.95 (0.77-1.2) | 0.64 |
|  | *Birth Weight | 781 |  | 3290 (2930-3650) | 0 | 0.0% | 511 |  | 3260 (2910-3620) | 577 | 53.0% | 0.99 (0.99-1.0) | 0.28 | 1.0 (0.99- 1.0) | 0.41 |
|  | *Gestational Age (wks) | 781 |  | 39 (38-40) | 0 | 0.0% | 523 |  | 39 (38-40) | 565 | 51.9% | 1.1 (0.98-1.2) | 0.13 | 1.0 (0.93-1.1) | 0.67 |
| **Neonatal** | Days in the NICU | 658 |  | 3 (2-12) | 123 | 15.7% | 456 |  | 4 (2-14) | 632 | 58.1% | 1.0 (0.99-1.0) | 0.89 | 1.0 (0.99-1.0) | 0.88 |
|  | Hyperbilirubinemia | 738 | 7.7% |  | 43 | 5.5% | 1088 | 5.6% |  | 0 | 0.0% | 0.73 (0.5-1.1) | 0.11 | 0.84 (0.57-1.3) | 0.47 |
| **Post-neonatal** | Post-neonatal brain injury | 721 | 9.2% |  | 60 | 7.7% | 519 | 7.7% |  | 569 | 52.3% | 0.83 (0.53-1.3) | 0.37 | 0.90 (0.59-1.4) | 0.63 |
|  | Age at MRI | 620 |  | 14.4 (5.0-27.4) | 161 | 20.6% | 486 |  | 19 (3.6-36.2) | 602 | 55.3% | 1.0 (1.0-1.0) | 0.002 | 1.0 (0.99-1.0) | 0.13 |
|  | Surgery or Botox | 712 | 59.4%% |  | 69 | 8.8% | 541 | 58.4% |  | 547 | 50.3% | 0.96 (0.76-1.2) | 0.72 | 0.95 (0.74-1.2) | 0.72 |
|  | Gastrostomy /Jejunostomy | 732 | 12.3% |  | 49 | 6.3% | 617 | 12.0% |  | 471 | 43.3% | 0.97 (0.69-1.4) | 0.87 | 1.1 (0.70-1.8) | 0.48 |
| **Comorbid conditions** | Gavage feeds | 737 | 6.4% |  | 44 | 5.6% | 618 | 3.7% |  | 470 | 43.2% | 0.57 (0.33-0.97) | 0.03 | 0.61 (0.34-1.1) | 0.11 |
|  | Cognitive Impairment | 517 | 54.2% |  | 264 | 33.8% | 331 | 56.2% |  | 757 | 69.6% | 1.1 (0.81-1.4) | 0.56 | 0.98 (0.68-1.4) | 0.84 |
|  | Visual Impairment | 706 | 19.8% |  | 75 | 9.6% | 538 | 15.6% |  | 550 | 50.6% | 0.75 (0.55-1.0) | 0.06 | 0.76 (0.53-1.1) | 0.13 |
|  | Eyeglasses | 653 | 19.8% |  | 128 | 16.4% | 527 | 23.2% |  | 561 | 51.6% | 1.2 (0.92-1.6) | 0.16 | 1.1 (0.88-1.6) | 0.2 |
|  | Auditory Impairment | 703 | 7.8% |  | 78 | 10.0% | 531 | 8.3% |  | 557 | 51.2% | 1.1 (0.69-1.6) | 0.77 | 0.98 (0.63-1.51) | 0.81 |
|  | Words | 490 | 42.5% |  | 291 | 37.3% | 339 | 41.6% |  | 749 | 68.8% | 0.97 (0.72-1.3) | 0.81 | 1.0 (0.71-1.5) | 0.99 |
|  | Words and Non-verbal system | 490 | 18.6% |  | 291 | 37.3% | 339 | 17.4% |  | 749 | 68.8% | 0.92 (0.63-1.3) | 0.67 | Words used |  |
|  | Non-verbal system only | 490 | 12.9% |  | 291 | 37.3% | 339 | 19.2% |  | 749 | 68.8% | 1.6 (1.1-2.4) | 0.01 |  |  |
|  | No Communication | 490 | 22.5% |  | 291 | 37.3% | 339 | 15.6% |  | 749 | 68.8% | 0.64 (0.43-0.93) | 0.02 |  |  |
| **Severity** | GMFCS Severity at ≥ 5 years | 634 |  | 1 (1-4) | 147 | 18.8% | 456 |  | 2 (1-4) | 632 | 58.1% | 1.1 (0.99-1.2) | 0.07 | 1.1 (0.95-1.4) | 0.13 |
|  | MACS Severity at ≥ 5 years | 540 |  | 2 (1-4) | 241 | 30.9% | 392 |  | 2 (1-4) | 696 | 64.0% | 1.0 (0.93-1.1) | 0.65 | 0.95 (0.80-1.1) | 0.47 |
|  | Non-Ambulatory CP | 634 | 26.0% |  | 147 | 18.8% | 456 | 29.0% |  | 632 | 58.1% | 1.2 (0.88-1.5) | 0.29 | 0.81 (0.41-1.6) | 0.53 |

*denotes variables in the CP risk calculator
