# Supplementary material for: Cerebral palsy characteristics in term‐born children with and without detectable perinatal risk factors: A cross‐sectional study
Source: Dev Med Child Neurol. 2024 Oct 15;67(4):475–85. doi: 10.1111/dmcn.16111 (PMC11875524; doi:10.1111/dmcn.16111)
Supplement: Supplementary file 3 — Table S3: Results of 12 variables on which the CP risk calculator is based. [file DMCN-67-475-s002.docx]

Etable 3: Results of 12 variables on which the CP risk calculator is based

|  |  | **Probability ≤ 0.3 (n = 219)** | | **Probability > 0.3 (n = 562)** | | **Univariable** | |
| --- | --- | --- | --- | --- | --- | --- | --- |
|  |  | **N** | **Missing no./Total No. (%)** | **N** | **Missing no./Total No. (%)** | **OR (95% CI)** | **P-Value (Fisher's Exact Test)** |
| **Maternal/Pregnancy** | ***No. of pregnancies** | 2 (1 - 2) | 0/219 (0) | 2 (1 - 3) | 0/562 (0) | 0.59 ().51-0.69) | <0.001 |
|  | ***No. of Miscarriages** | 0 (0 - 0) | 0/219 (0) | 0 (0 - 1) | 0/562 (0) | 0.83 (0.66-1.1) | 0.13 |
|  | ***Gestational Diabetes** | 3/219 (1.4%) | 0/219 (0) | 75/562 (13.4%) | 0/562 (0) | 0.09 (0.02-0.28) | < 0.001 |
|  | ***Preeclampsia** | 0/219 (0%) | 0/219 (0) | 34/562 (6.1%) | 0/562 (0) | N/A |  |
|  | ***Chorioamnionitis** | 0/219 (0%) | 0/219 (0) | 80/562 (14.2%) | 0/562 (0) | N/A |  |
|  | ***Tobacco** | 8/219 (3.7%) | 0/219 (0) | 123/562 (21.9%) | 0/562 (0) | 0.14 (0.056-0.28) | < 0.001 |
|  | ***Drugs** | 0/219 (0%) | 0/219 (0) | 30/562 (5.3%) | 0/562 (0) | N/A |  |
| **Delivery** | ***Prolonged Rupture of Membranes** | 13/219 (5.9%) | 0/219 (0) | 37/562 (6.6%) | 0/562 (0) | 0.90 (0.42-1.8) | 0.74 |
|  | ***Apgar at 5 min** | 9 (9 - 10) | 0/219 (0) | 9 (6 - 9) | 0/562 (0) | 4.3 (3.2-5.9) | <0.0001 |
| **Infant** | ***Male** | 105/219 (48.0%) | 0/219 (0) | 342/562 (60.9%) | 0/562 (0) | 0.59 (0.43-0.82) | 0.0011 |
|  | ***Birth Weight** | 3430 (3175 - 3740) | 0/219 (0) | 3220 (2820 - 3601.25) | 0/562 (0) | 1.0 (1.0-1.0) | <0.0001 |
|  | ***Gestational Age (wks)** | 40 (39 - 40) | 0/219 (0) | 39 (38 - 40) | 0/562 (0) | 1.7 (1.5-1.9) | <0.001 |
