# Supplementary material for: Cerebral palsy characteristics in term‐born children with and without detectable perinatal risk factors: A cross‐sectional study
Source: Dev Med Child Neurol. 2024 Oct 15;67(4):475–85. doi: 10.1111/dmcn.16111 (PMC11875524; doi:10.1111/dmcn.16111)
Supplement: Supplementary file 4 — Table S4: Province of birth for participants. [file DMCN-67-475-s003.docx]

Etable 4: Province of birth for participants

| Birth Province | Undetectable CP risk (n=195) | | Detectable CP risk (N=586) | |
| --- | --- | --- | --- | --- |
| Alberta | 51 | 26.2% | 185 | 31.8% |
| British Columbia | 3 | 1.5% | 24 | 4.1% |
| Newfoundland | 1 | 0.5% | 9 | 1.5% |
| Northwest Territories | 0 | 0.0% | 1 | 0.2% |
| Nova Scotia | 1 | 0.5% | 7 | 1.2% |
| Ontario | 6 | 3.1% | 40 | 6.9% |
| Quebec | 132 | 67.7% | 314 | 54.0% |
| Saskatchewan | 1 | 0.5% | 2 | 0.3% |
| Missing | 0 | 0.0% | 4 | 0.7% |
